# Loop Suspension in Fractures of the Thigh

**Published:** 1918-03

**Authors:** 


**Loop Suspension in Fractures of the Thigh.** By HEITZ-BOYER and POULIQUEN, Surgeons of the IVth Army. Trans. and Abs. from the *Bulletins et Mémoires de la Société de Chirurgie de Paris*, Oct. 16, 1917.

The authors have found suspension by bronze wires the most successful means of anterior reduction for the inferior fragment of a femur in cases in which that fragment tends to drop below its proper level. In some cases they have found it desirable to apply the wire loops to both bone fragments. The wire may be looped around the end of the bone, if necessary, by means of a curved needle. The ends of the loop, projecting outside the skin, may be suspended by various contrivances attached to the frames now in use for reduction and suspension. It is seldom necessary for the wire to penetrate the bone to insure stability, although in extreme cases it may be so applied.

The authors have used this method chiefly for vertical reductions, but they have had success in a few cases where oblique or even longitudinal reduction was necessary. They do not feel prepared, as yet, to recommend this method for longitudinal reductions in general.

The wires have been left in place for as long as 45 days. At the end of this time they could be removed easily without anesthesia. In no case did they cause infection.

